# Flaxseed Bioactive Compounds: Chemical Composition, Functional Properties, Food Applications and Health Benefits-Related Gut Microbes

**DOI:** 10.3390/foods11203307

**Published:** 2022-10-21

**Authors:** Abdul Mueed, Sahar Shibli, Sameh A. Korma, Philippe Madjirebaye, Tuba Esatbeyoglu, Zeyuan Deng

**Affiliations:** 1 State Key Laboratory of Food Science and Technology, Nanchang University, Nanchang 330047, China; 2 National Agriculture Research Center, Food Science Research Institute, Islamabad 44000, Pakistan; 3 Department of Food Science, Faculty of Agriculture, Zagazig University, Zagazig 44519, Egypt; 4 School of Food Science and Engineering, South China University of Technology, Guangzhou 510641, China; 5 Department of Food Development and Food Quality, Institute of Food Science and Human Nutrition, Gottfried Wilhelm Leibniz University Hannover, Am Kleinen Felde 30, 30167 Hannover, Germany

**Keywords:** bioactive compound, biological activity, food application, functional properties, gastroenterology, gut microbes, *Linum usitatissimum* L., metabolism, superfood, veganism

## Abstract

Flaxseed (*Linum usitatissimum* L.) has gained worldwide recognition as a health food because of its abundance in diverse nutrients and bioactive compounds such as oil, fatty acids, proteins, peptides, fiber, lignans, carbohydrates, mucilage, and micronutrients. These constituents attribute a multitude of beneficial properties to flaxseed that makes its use possible in various applications, such as nutraceuticals, food products, cosmetics, and biomaterials. The importance of these flaxseed components has also increased in modern times because of the newer trend among consumers of greater reliance on a plant-based diet for fulfilling their nutritional requirements, which is perceived to be hypoallergenic, more environmentally friendly, sustainable, and humane. The role of flaxseed substances in the maintenance of a healthy composition of the gut microbiome, prevention, and management of multiple diseases has recently been elucidated in various studies, which have highlighted its importance further as a powerful nutritional remedy. Many articles previously reported the nutritive and health benefits of flaxseed, but no review paper has been published reporting the use of individual flaxseed components in a manner to improve the techno-functional properties of foods. This review summarizes almost all possible applications of flaxseed ingredients in food products from an extensive online literature survey; moreover, it also outlines the way forward to make this utilization even better.

## 1. Introduction

Flaxseed (*Linum usitatissimum* L.), commonly known as flaxseed or linseed, is an annual crop mainly grown for oil, fiber, food, and feed purposes. The significance of this crop has greatly increased in the modern world because of its exceptional nutritive content with a strong biological activity that has made its use possible in various applications such as functional foods, health supplements, and skincare products [1,2,3]. The importance of flaxseed has also recently increased because of the newer trend of veganism among consumers all over the world and the number of social, ethical, religious, moral, environmental, and sustainability concerns associated with the consumption of animal-based products. This review paper focuses on the techno-functional properties of individual flaxseed components in foods that have never been reported in a comprehensive manner in one place before [4]. Flaxseed is becoming increasingly famous as a superfood because of its beneficial role in regulating gut flora and alleviating symptoms of many human diseases, such as cardiovascular ailments, diabetes, neural disorders, menopause, skin problems, gastrointestinal issues, and even cancers [5]. Furthermore, the proteins and cyclic peptides of flaxseed have been found to possess preferable antioxidant, antihypertensive, anti-inflammatory, immuno-suppressive, and anti-diabetic properties [6].

The inclusion of flaxseed in a diet as an important grain has been recently emphasized due to the nutritive benefits from its constituents, particularly fats, proteins, lignans, and fiber, and their use in the development of various value-added products [7]. The functional property of a food component is the behavior based on its biochemical composition, which confers specific sensory characteristics both during processing and storage when utilized as a food additive. For instance, flaxseed mucilage (FM) possesses a potent water-binding ability, which has been successfully utilized in foods to enhance the stability, viscosity, and consistency of beverages [8]. In addition, FM also has prebiotic properties, which are the ability to bulk up the stools and regulate the beneficial composition of microbes inside the gut that ultimately reduces the symptoms of constipation and irritable bowel syndrome (IBS) [6]. We focused on the chemistry of bioactive compounds, functional properties, and food applications, as well as the role of flaxseed bioactive compounds in the maintenance of beneficial intestinal microbiomes. However, the industrialization of flaxseed and its bioactive compounds has not been made possible yet as there are constraints to their large-scale production and evaluation of their efficacy on a real-time basis.

## 2. Nutritional Composition of Flaxseeds

### 2.1. Lipids

**Figure 1:**
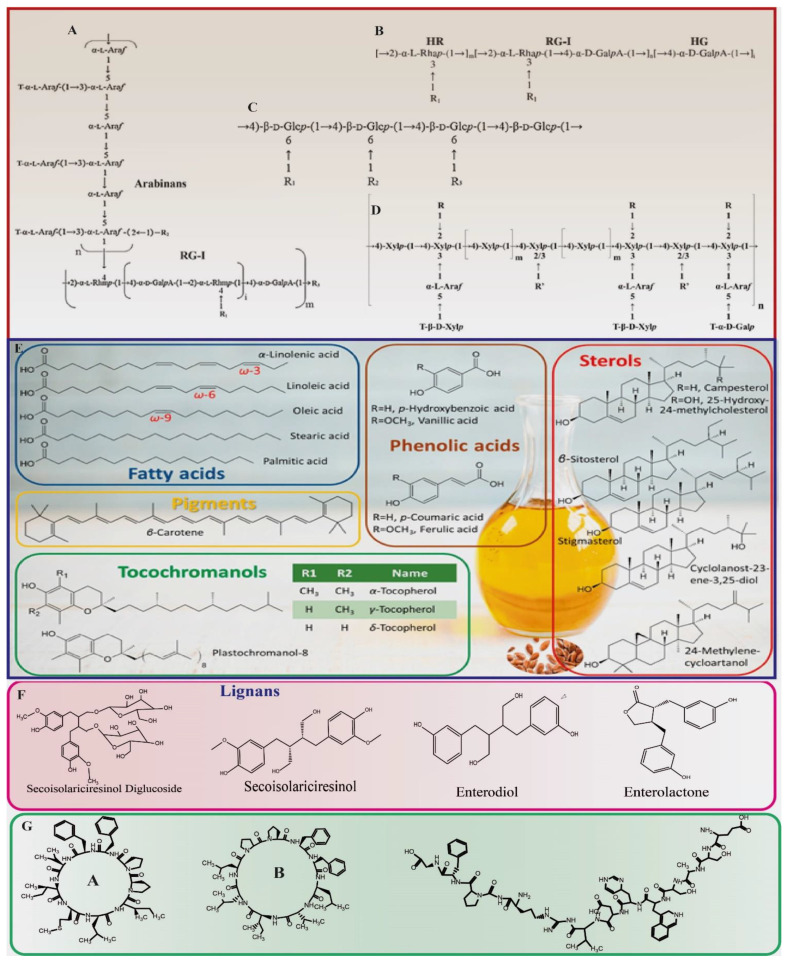
Overview of flaxseed bioactive compounds. (**A**) Proposed structure of KPI-ASF as RG-1 bridge-linked arabinans. (**B**) Proposed repeating unit of the acidic fraction gum (HR, RG-1, and HG refer to homorhamnan, rhamnogalacturonan-I, and homogalacturonan, respectively). (**C**) Proposed structure of KPI-EPF as xyloglucans. (**D**) Proposed structure of FM-NFG as arabinoxylans. (**E**) Flaxseed oil, phenolic acids, sterols, pigments, and tocochromanols. (**F**) Lignans and their metabolites. (**G**) Cyclolinopeptide-A, cyclolinopeptide-B, and alcalase-derived antioxidant peptide.

Flaxseed oil (FO) is divided into monounsaturated, polyunsaturated, and saturated fractions on the basis of fatty acid components (Figure 1) [9]. It is mainly abundant in total unsaturated fatty acids (87.8–89.8%) in comparison with the small amount of saturated fatty acids [10]. An investigation of FO extracted with petroleum ether elucidated *α*-linolenic (C18:3, *ω*-3, 42.4%), linoleic (C18:2, *ω*-6, 26.2%), palmitic (C16:0, 12.9%) and stearic acids (C18:0, 10.7%) as the major constituents [11]. Previous studies, however, revealed a little higher amount of *α*-linolenic (ca. 49–53%) and oleic (C18:1, *ω*-9, ca. 16–21%) acids along with a lower linoleic acid level (ca. 15–17%) and ascribed this variation to the difference in environment and farming conditions [12,13]. On the other hand, when *n*-hexane was used as an extracting solvent by Ishag and Khalid [11], it produced contrasting results with greater linoleic acid (46.5%) and lower *α*-linolenic acid (11.6%), while 18.0% palmitic acid in FO. It has been revealed that *α*-linolenic acid contents of flaxseed varieties from New Zealand and Canadian origin approximate 60%, which is far greater than the varieties belonging to Pakistan, Ethiopia, Egypt, and the USA. This increase is attributed to the cool, humid environment and optimal cultural practices (Table 1) [10,14].

Alpha-linolenic acid (ALA) is an essential polyunsaturated fatty acid with omega carbon at position three, which cannot be synthesized by the human body itself. ALA is used in the synthesis of docosahexaenoic acid (DHA) and eicosapentaenoic acid (EPA) through different biosynthetic pathways, which are required for the normal growth, development, and maintenance of the human body, especially the brain and skin. Flaxseed oil is a very important source of ALA, but it has been employed to a limited extent for human benefits because of its low conversion ratio to DHA and EPA, which is a big hurdle faced by the scientific community these days [15]. The bioavailability of ALA is also dependent on the form of flaxseed consumed. For example, it is greater in flaxseed oil than in its milled form or whole seed. Additionally, the high unsaturated fatty acid content of flaxseed makes it highly prone to oxidative damage during processing stages, which is another threat that needs to be addressed to take maximum advantage of its nutritional contents [15]. Moreover, another study reported an appreciable amount of phospholipids, including phosphatidylethanolamine (27–40%), phosphatidylinositol (29–32%), phosphatidylcholine (7–18%), lysophosphatidylcholine (8–21%), phosphatidylglycerol (1–4%) and phosphatidic acid (1–9%), along with a small amount of palmitic acid (about 5%) and stearic acid (about 3%) in the lipid portion of flaxseed [14].

**Table 1:** Protein, oil, and phenolic acid composition of flaxseed.

**Composition of protein [16,17]**		
**Amino acids**	**Flaxseed protein meal (g/100 g)**	**Flaxseed protein** **hydrolysate (mg/g)**
Alanine	4.59	n.a *
Arginine	10.63	33.12
Asparagine	9.76	496.51
Cysteine	3.80	n.a *
Glutamic acid	26.92	911.05
Glycine	6.14	288.43
Histidine	2.45	118.63
Isoleucine	5.21	207.49
Leucine	6.82	261.79
Lysine	4.18	191.41
Methionine	2.20	104.09
Phenylalanine	5.33	284.49
Proline	5.24	n.a *
Serine	5.88	n.a *
Threonine	4.19	169.94
Tryptophan	1.38	n.a *
Tyrosine	2.94	500.33
Valine	5.17	131.43
**Composition of oil (%) [15]**		
**Fatty acids**	**Flaxseed oil**	**Soybean oil**
Myristic (C14:0)	0.03–0.05	n.a *–0.12
Pentadecanoic acid (C15:0)	n.a *–0.01	n.a *
Palmitic (C16:0)	4.58–6.42	10.80–11.50
Palmitoleic (C16:1)	0.04–0.20	n.a *–0.16
Margaric (C17:0)	n.a *–0.04	n.a *–0.04
Margaroleic (C17:1)	n.a *–0.03	n.a *
Stearic (C18:0)	3.65–5.96	3.62–4.11
Oleic (C18:1n-9)	16.33–22.56	20.80–23.50
Linoleic (C18:2n-6)	9.18–15.88	50.23–53.33
Linolenic (C18:3n-3)	42.97–61.06	6.76–7.65
Arachidic (C20:0)	0.01–0.20	n.a *–0.32
Gadoleic (C20:1)	n.a *–0.21	n.a *–0.22
Eicosanoic (C20:2)	n.a *–0.09	n.a *
Behenic (C22:0)	0.11–0.14	n.a *–0.27
Lignoceric (C24:0)	0.04–0.13	n.a *–0.13
Saturated	8.42–12.90	14.42–16.18
Total Monounsaturated	16.37–23.00	20.8–23.88
Total Polyunsaturated	52.15–76.94	56.99–60.98
**Composition of phenolic acids and lignans (mg/100 g) [16,18]**		
	**Non-defatted extracts**	**Defatted extracts**
*p*-Hydroxybenzoic acid	1719	6454
Chlorogenic acid	720	1435
Ferulic acid	161	313
Coumaric acid	87	130
Gallic acid	29	17
Vanillic acid	22	42
Sinapic acid	18	27
Protocatechuic acid	7	7
Caffeic acid	4	15
Diphyllin	4.2	n.a *
Secoisolariciresinol diglucoside	1300	n.a *
Secoisolariciresinol	156	n.a *
Laricinesol	1.7	n.a *
Matairesinol	3.1	n.a *
Pinoresinol	0.8	n.a *
**Composition of vitamins and pigments (µg/g) [19,20]**		
	**Raw flaxseed**	**Boiled flaxseed**
α-Tocopherol	6.26	4.56
β-Tocopherol	1.07	0.89
γ-Tocopherol	302.0	256.2
δ-Tocopherol	2.26	2.09
β-Carotenoid	0.52	0.48
Xanthophyll	27.1	20.2
**Sprouted**	**LT001 ^a^**	**Zhongya 4 ^a^**
Lutein	56.03	8.14
Zeaxanthin	2.38	2.76
β-Cryptoxanthin	1.64	1.86
ε-Carotene	1.44	1.61
β-Carotene	5.18	5.87

* n.a = data not available, ^a^ = variety.

The total sterol content of FO ranged from 4720 to 7550 mg/kg oil [21], from which sitosterol made the most quantity averaging almost 240 mg/100 g while campesterol and stigmasterol were other significant constituents averaging about 110 and 50 mg/100 g in corresponding order [22]. FO also contained significant quantities of 24-methylenecycloartanol, 25-hydroxy-24-methylcholesterol and cyclolanost-23-ene-3,25-diol [21]. Tocochromanols are another category of strong antioxidant compounds present in flaxseed oil, which besides being amphipathic, also possesses vitamin E activity ranging from 154–934 mg/kg by virtue of particular substances commonly called tocopherols and tocotrienols [23]. A huge variation in the vitamin E activity of FO was observed due to the difference in plant variety, location, growing, extraction, and storage conditions. Furthermore, this dual characteristic of tocochromonals for being hydrophilic and hydrophobic at the same time comes from a tyrosine-derived polar moiety and a poly-prenyl side chain, respectively. Tocotrienols are tocochromanols with a geranyl-geranyl side chain, while tocopherols possess a phytyl-side chain [24].

The total tocopherol content in Pakistani and Egyptian cultivars was greater than that of Canadian and American cultivars [10], which is an indication of their better antioxidant potential. Tocopherols are the fat-soluble vitamins (460–610 mg/kg) found in the greatest amount in FO, trailed by plastochromanol-8 (270–370 mg/kg), which is a functional analog of *γ*-tocotrienol and lastly *α* tocopherol that averages about 1–8 mg/kg [25]. The study also revealed that the *γ*-tocotrienol and plastochromanol-8 had a positive correlation, while *δ*-tocopherol had a negative correlation with temperature and amount of sunlight received during seed ripening. The cause behind this biological mechanism needs to be sought out for taking advantage of this relationship for human use [10].

### 2.2. Polysaccharides

FM is separated into two types of polysaccharide fractions on the basis of the net charge that is acidic and neutral. Arabinoxylans with β-D-(1,4)-xylan make the backbone of a neutral fraction, which is also free of uronic acid, while the acidic fraction is mainly composed of sugars, which are building blocks of pectic substances such as galactose, rhamnose, and galacturonic acid (Figure 1) [26]. The analysis of the chemical composition of FM from different genotypes showed that FM from yellow seeds had lower galacturonic acid (13–16%) and rhamnose (12–14%) contents, while higher neutral sugars like xylose (39–48%) content in contrast to FM obtained from brown seeds [27]. Moreover, it has been documented that the neutral fraction of FM with high molecular weight (MW; 1470 kDa) also contains uronic acids in a small amount (1.8%), which gives it a pseudo-plastic flow behavior [28].

The acidic fraction of FM is mostly constituted by two sub-fractions of rhamnogalacturonans, one with greater MW (1510 kDa) and the other with a lesser MW (341 kDa). The structure of rhamnogalacturonan from flaxseed hull was explicated through methylation analysis and 1D/2D NMR spectroscopy which showed it to be a structure consisting of rhamnogalacturonan-1 (RG-1) structure with diglycosyl repetition unit →2)-α-L-Rhap-(1→4)-α-D-GalpA-(1→as depicted in Figure 1 [29]. Six types of RG-1 from FM were obtained from ion-exchange chromatography and a light scattering detector [30]. Furthermore, MWs of acidic fractions were also determined through size exclusion chromatography as follows 756.4 kDa, 718.8 kDa, 505.6 kDa, 457.5 kDa, 354.8 kDa, and 593.2 kDa. However, the rhamnose to galacturonic acid ratio (1.22 to 0.85) and degree of branching (0.33 to 0.65) of the acidic fractions varied considerably. It was also revealed that RG-1 blocks are often singly substituted with sugars like galactose, fructose, rhamnose, or sometimes short, neutral monosaccharides. Polysaccharides in FM, when dissolved in water, attained random coil confirmation, the MWs of which fluctuated between 1.6 × 10^6^ and 1.0 × 10^7^ g/mol, whereas, in the salt solution, they adopted more regular, spherical, closed shapes, which differed in weight from the spiral and close confirmations MWs of which varied from 1.5 × 10^6^ to 4 × 10^8^ g/mol [31].

### 2.3. Protein/Peptides

Flaxseed is an abundant source of proteins, which make up to 23% of the total seed weight, and this amount increases to 35 to 40% in meal after oil extraction. A balanced amino acid combination of flaxseed gives it a high protein quality score (82%), which is even better than that of soybean [32]. Similarly, the lysine to arginine ratio of flaxseed of 0.37 is far lesser than that of soybean (0.88), which is indicative of its lower lipidemic and atherogenic potential and, thus, heart friendliness (Table 1) [32]. Flaxseed mainly contains two types of proteins, namely albumins and globulins, on the basis of solubility properties, which are also known as linins and colinins. Globulins make up 80% of total proteins. They consist of subunits with a high MW ranging between 252 and 298 kDa (18.6% nitrogen; 11–12S) and smaller percentages of alpha-helical (3%) and beta structures (17%) [33,34].

Flaxseed is considered a preferable source of protein because of the appreciable amounts of sulfur-based amino acids, such as cysteine and methionine; branched-chain amino acids, such as leucine, isoleucine, aline, and essential amino acids, such as tyrosine, threonine, and lysine. Flaxseed is rich in storage proteins such as aspartic acid, glutamine, asparagine, and arginine, like other seeds, which contribute to its high amide content [32]. Madhusudhan and Singh [33] isolated flaxseed globulins (FG) through SDS-PAGE in their investigation. It was found that FG contained five subunits with MW lying between 11 and 61 kDa and six subunits with MW lying between 41 and 55 kDa linked with disulfide bridges. When treated with mercaptoethanol, the larger subunits with MWs from 50 to 55 kDa disassociated into one acidic (40 kDa) and one basic smaller subunit (20 kDa). Likewise, five types of globulins were sorted in another study on the basis of MWs from flaxseeds with MWs of 14.4, 24.6, 30.0, 35.2, and 50.9 kDa, among which acidic subunits were a little bigger than basic subunits [35]. A large protein fraction (365 kDa) was isolated from defatted and dehulled flaxseed through anion-exchange chromatography, which was later separated into three more fractions (20, 23, and 31 kDa) when subjected to reducing SDS-PAGE. Four subunits of 11S globulin were identified from FP, which consisted of a pair of α- and β-chains linked with disulfide bonds [32]. A small amount of 7S globulin subunits (21 to 54 kDa) were also identified, along with other low MW (7 to 10 kDa) fractions.

Conlinins are a type of albumin found in the seeds of many plant species. It consists of a polypeptide chain with a 16–18 MW and a 1.6–2 sedimentation coefficient. These proteins have more organized confirmation due to greater disulfide bonds, which are overall composed of 26% α-helices and 32% β-structures [32]. Furthermore, these albumins are rich in lysine, arginine, cysteine, glutamine, and alanine [36]. Flaxseed is an important source of another group of proteins or peptides called cyclolinopeptides, orbitides, or linosurbs. There are more than 25 kinds of these compounds that have been distinguished [6]. Orbitides mostly consist of 8–10 amino acids. The structure of one famous Cyclolinopeptide-A is as follows: Pro-Pro-Phe-Phe-Leu-Ile-Ile-Leu-Val. These compounds have been found to possess multiple beneficial traits, such as being immunosuppressive, anti-malarial, anti-tumor, and a protectant against bone degeneration. Linosurbs are usually cyclic, hydrophilic in nature, linked with N-C bonds, and are named on the basis of the first amino acid or prolyl residue in the protein sequence. Orbitides are also present in many other plant species and are used for the chemical synthesis of methionine sulfones, alcohol, and acetonitrile solvates, which are useful for many health and biomedical uses [37].

### 2.4. Phenolic Compounds and Carotenoids

Phenolic substances possess numerous health advantages. Flaxseed has a variety of phenolic compounds, which are divided into two categories, namely phenolic acids and lignans (Table 1) and (Figure 1). The range of phenolic acids in a Canadian flaxseed variety was found to be varying between 790–1030 mg/100 g, out of which chlorogenic acid, *p*-hydroxy benzoic acid, ferulic acid, vanillic acid, and coumaric acid make the highest portion, while lignans, namely matairesinol, pinoresinol, diphyllin, and secoisolariciresinol made a smaller portion [16,38]. Lignans are low molecular weight phenolic dimers comprising of 2,3-dibenzylbutane as a base structure. They are mostly present in the outer coat of the seed [16]. Secoisolariciresinol diglucoside (SDG) is a major lignan in flaxseed averaging about 610–1300 mg/100 g [39]. Lignans are powerful anti-oxidant substances in flaxseed, which has made them the center of attention for many studies in recent times [40]. The phenolic acid composition of flaxseed before and after fat extraction is presented in Table 1, which shows that whole seeds have a lesser phenolic acid content than meal after oil extraction. The maximum recovery ratio of lignans was obtained by Gutiérrez and Rubilar [41] with 50% ethanol, 1:60 solid-to-liquid ratio, 30 min shaking, 200 rpm speed, and at 25 °C.

Carotenoids are organic compounds with 40-carbon atoms in many seeds and fruits, which gives them red, orange, and yellow colors, besides being precursors of vitamin A [42]. β-Carotene is one such important pigment that has the highest pro-vitamin A activity [43]. The carotenoid content of flaxseed was found to be 0.7–3.1 mg/kg; however, Farag and Elimam [44] reported a far greater amount of β-carotene in flaxseed oil (77 mg/kg). Furthermore, there is a positive correlation between the amount of tocochromanol and carotenoid levels in flaxseed and the number of sunshine hours received during the seed maturation phase. Carotenoids play a crucial role against photo-oxidation, and thus it holds special importance with reference to the high unsaturated lipid content of flaxseed.

## 3. Food Applications of Flaxseed and Its Components

### 3.1. Flaxseed Kernel

The consumption of flaxseed has been emphasized in recent times owing to its good nutritional value, particularly the high amount of healthy fats, proteins, and lignans. Flaxseed is available for human utilization in four forms, including oil, whole seeds, ground, and defatted oilseed meal [45]. Commercially, flaxseed is being widely used in packaged food products worldwide, for example, in cereals, bread, biscuits, fortified bars, soups, snacks, etc., due to the same functional benefits (Table 2) [16].

**Figure 2:**
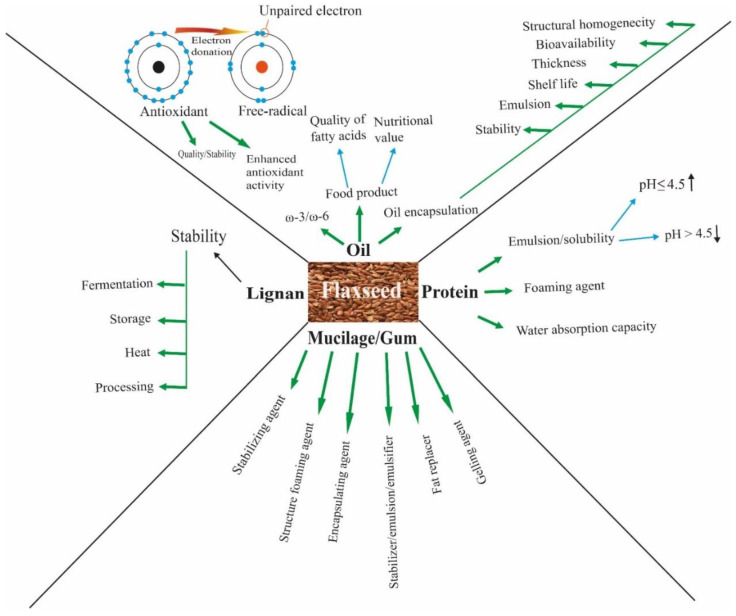
Functional properties of flaxseed bioactive compounds.

There is a growing demand among customers for plant-based proteins due to their health-promoting effects, in addition to the broad range of social concerns associated with the consumption of animal products [83]. Therefore, flaxseed and its derived products are gaining significance as an important dietary constituent due to their favorable lipid composition, high protein, lignan, and balanced amino acid contents. Apart from the nutritional value, flaxseed ingredients have the ability to enhance the functional characteristics of food products, such as emulsifying, foaming, and viscosity properties. It also has the potential to be utilized as an alternative bulk protein ingredient and thickener in sauces and salad dressings, replacing egg, with the added advantage of lignan and fiber components (Figure 2) [16]. Flaxseed flour, when added to wheat-based baked goods after roasting, improved the physical strength of the dough by increasing its water absorption, binding ability, protein content, and crumb softness and reduced the overall carbohydrate contents [72,76]. However, when the level of supplementation exceeded 20%, it negatively affected the textural properties of wheat bread, such as crumb softness, extensibility, color, and volume, by interfering with gluten development [53,75].

The consumer does not prefer any food that is not palatable and acceptable in terms of color, appearance, taste, aroma, and texture qualities. Several characteristics of flaxseed can offset the flavor profile of foods where it is utilized. For example, high oil content in products can make them susceptible to oxidation or rancidity, which eventually leads to the development of a bitter taste. The addition of flaxseed up to a certain level does not negatively impact the food’s flavor [53,72]. However, flaxseed, when added in significant quantities to the foods, such as bagels, muffins, and meat products, gives them a characteristic nutty flavor and aroma, which reduces their overall acceptability [47,52,84]. Flaxseed milk has been launched in the market as an alternative to animal-derived milk with some added advantages, such as high ALA content, zero cholesterol, and lactose content, along with ease of consumption and preservation [5]. Another interesting prospect of flaxseed is that it retards the growth of some food spoilage microorganisms [78]. Supplementation of flaxseed as an ingredient in many food formulations, such as bread, muffins, pasta, pork, meats, etc., was found to extend not only the shelf life of food, but also contributed toward their nutritional and functional benefits depending upon the composition, addition level, and processing methods [85].

### 3.2. Flaxseed Oil

Flaxseed oil has gained popularity among buyers worldwide due to its plentiful nutritional advantages and food applications. However, the susceptibility of flaxseed oil to oxidative degeneration is the main limiting factor, which limits its use in foods and other applied purposes. Earlier research has shown that some natural antioxidants can slow down the oxidation process of flaxseed oil and prevent it from rancidity (Figure 2). Condori and Chagman [86] reported that lycopene can significantly enhance the antioxidant capacity of flaxseed oil by reducing the storage stability degradation kinetics by 42%, which in turn prolonged shelf life by 31%.

The use of flaxseed in a nano-emulsion or microencapsulated form has been found to significantly enhance the bioavailability of ω-3 polyunsaturated fatty acids. These technologies are frequently being used by the food and health industry in modern times for the protection of sensitive ingredients from heat, light, oxygen, water, and digestive processes and thus making possible their delivery at target sites in an effective manner [87]. Flaxseed components are mostly spray- or freeze-dried and sometimes are also subjected to complex aggregation or coacervation before being encapsulated, which further extends their shelf life [15]. Milk proteins, particularly casein and whey, were found to be better emulsifying agents for polyunsaturated fatty acids derived from flaxseed than commonly used surfactants Tween and Citrem when equated for antioxidative ability and tolerance through in vitro digestion testing schemes [88].

Nano-emulsion technology was also analyzed for the encapsulation of flaxseed oil. It not only provided protection against heat, oxidative deterioration, and nutritive losses but, in combination with natural antioxidants, also was a good replacement for synthetic antioxidants, which are considered extremely harmful to human health. Nano-emulsions were thus proven to form a dispersion of flaxseed components with favorable stability, solubility, rheology, and non-toxic characteristics [89].

Nasrabadi and Goli [90] formulated composite particles of flaxseed proteins (FP) and soluble portions of flaxseed mucilage (SFM) and analyzed their effect on the stability of Pickering emulsions. The composite particles (FP-SFM) withstood the storage stresses and attributed to the stability and solubility of emulsions in a better manner than when flaxseed proteins were utilized alone. Therefore, these composite particles (FP-SFM) provide promising future prospects for the encapsulation of oleophilic and phytoactive substances for their utilization in food and pharmaceutical preparations [15].

Another study was conducted in pursuit of a plant-based antioxidant system, where pea protein and tannic acid complexes were investigated for providing stability to flaxseed emulsions against oxidative rancidity. These aggregates not only attributed a strong antioxidant ability to oil emulsion but also bore the stresses of gastric digestion; thus, they can be a good carrier of flaxseed emulsions in living organisms for achieving various nutraceutical uses [91]. Consequently, the selection of an appropriate emulsifier and technique for delivery has a crucial role in optimizing the bioavailability of a substance.

Flaxseed oil and its fractions were assessed in various studies to examine the effect of its supplementation on the quality of animal feed that ultimately improved the quality of animal products such as milk, meat, and eggs. When flaxseed-derived PUFA was added to broiler chicken feed along with selenium and vitamins E and C, it increased the amount of ω-3 fatty acids in poultry meat but also prevented it from oxidation at the carcass, storage, freezing, and cooking stages [92]. Likewise, when flaxseed oil was supplemented to the cows’ rations, it increased the milk yield as well as improved the percentage of functional fatty acids, specifically α-linoleic acid, conjugated linoleic acid, and vaccenic acid in the milk, while reducing the percentage of saturated fatty acids [93]. In another research by Moallem and Lehrer [94], the effect of α-linoleic acid addition from flaxseed was checked on the health of cows that were about to undergo parturition; it was revealed ALA improved milk yield, unsaturated fatty acids percentage, and fertility while decreased the incidence of ketosis, metritis, and mortality of calves.

Tilapia fish meat was evaluated for lipid composition after the inclusion of 7% flaxseed oil in their diet. It enhanced both the nutritional and physiological quality of meat but also affected its fatty acid composition in a favorable manner by increasing the proportion of n-3 fatty acids and lowering the proportion of saturated and n-6 fatty acids [95]. Furthermore, the potential of increasing ω-3 fatty acids in pork was reviewed by Huang and Chiba [96]. It was inferred that flaxseed lipids could improve the percentage of ALA and EPA in ham, which is beneficial for humans, but the rate of conversion of ALA to DHA was still found as a limitation that needs to be ameliorated in the future. Flaxseed oil was tested to produce a spreadable, low-trans-fat shortening with butter fat and palm stearin. As the percentage of flaxseed oil was increased from 2–10%, it caused a remarkable increase in α-linolenic acid percentage from 5.6–26% while at the same time significantly reduced the saturated acid content, trans-fat levels, and atherogenic index of the resulting shortening [97].

### 3.3. Protein

Flaxseed contains mainly two types of proteins called linins and colinins, which were first isolated by Vassel and Nesbitt [98] through a multistep extraction method including different reagents, namely petroleum ether, phosphate salt, glycol, sodium hydroxide, and final acidification with HCl to the isoelectric point (pI) which is 4.75 pH. All processing stages, such as dehulling, milling, and oil extraction, increased the amount, digestibility, and solubility of proteins, which was attributed to the removal of other nutritive constituents from the whole meal, such as the lipid portion [99]. The homogeneity of the flaxseed slurry depends upon the positive or negative charges on its proteins or repulsive forces among them, which maintains their solubility. However, when the pH of this matrix approaches near pI, the net charge on its proteins decreases, causing them to coalesce together, and thus they no longer retain their solubility. Flaxseed proteins are separated from the main slurry in this manner to be employed for various uses. The solubility of flaxseed proteins is also affected by heat; for example, the process of oil extraction reduced their solubility from 73.2 to 44.9%. Boiling similarly reduced the solubility of proteins, which was suggested to be caused by protein denaturation and the leaching of a non-protein-nitrogen fraction [16].

Flaxseed proteins possess some unique functional properties, which make them a preferred plant-based alternative to eggs or other animal-derived proteins. Firstly, flaxseed protein has a strong emulsification capacity (EC), due to which it can form a strong emulsion in many food systems by forming a coating around the oil droplets. The EC of flaxseed protein is significantly affected by heat, pH, and extraction conditions. The EC of alkali-solubilized flaxseed proteins was better than soy protein, gelatin, or whey protein. High heat caused structural damage to the flaxseed proteins, which reduced both their solubility and EC. Flaxseed proteins exhibited good EC in both acidic and basic conditions; thus, they can also be used as an emulsification agent in nano-emulsions for the delivery of bioactive compounds. The emulsification ability of FP got increased when they were used in combination with flaxseed mucilage, and it can be further enhanced by causing alterations in FP confirmations [100].

The second remarkable functional property of FP is its ability to form foam or foaming capacity that remains stable under a wide range of pH and temperatures. Plant foams perform better under acidic conditions. FP formed a very stable foam with a 25% volume increase from 2–6 pH. The FC increased further until pH 8, but the resultant foam was less stable. Similarly, the FC of flaxseed protein remained constant until 45 °C, but a marked increase in FC was noticed when the heat was between 45 and 80 °C. After the threshold of 80 °C, a progressive decline in FC was observed, which was attributed to protein denaturation and lowering of solubility. Furthermore, flaxseed produces foam with better stability than soy protein under all pH conditions [33,36].

Water and fat absorption capacity (AC) is another remarkable functional property that flaxseed proteins attribute to a food system. AC is the amount of water or fat that a protein can hold in a biological matrix without undergoing significant structural damage. This trait of FP originates from its polar amino acids, which play a significant role in holding a large amount of lipid content within flaxseed kernel while it also contributes to the good mouth feel and taste of food. The water and fat absorption capacity of flaxseed protein are even better than soy protein and gelatin. Flaxseed protein can be used as a meat extender owing to this property which can prevent the fat and weight loss of product during the cooking process [100].

### 3.4. Polysaccharide/Mucilage

Gum or mucilage is an important component of flaxseed, which is mostly located on the outer layers of the seed. It constitutes almost 8–10% of the whole seed weight [101]. FG attributes unique functional properties to foods owing to their unique polysaccharide composition, such as improving viscosity, emulsifying ability, rheology, and foaming ability. The hot water extraction method was found to be the best treatment for obtaining flaxseed gum that yielded polysaccharides with better stability, consistency, functional abilities, and commercial value. The properties of flaxseed gums vary on the basis of extraction methods and plant varieties. They can also be further altered through chemical or physical methods to achieve the desired purpose [26].

Cui and Mazza [102] reported that when FG was extracted at a high temperature from 85–90 °C with ion exchange chromatography, two fractions of FG gums were obtained with different properties, namely neutral fraction or arabinoxylans with higher viscosity and acidic fraction or rhamnogalacturonans with lesser viscosity due to smaller size polysaccharide units. Kaushik and Dowling [103] found that the temperature had a direct relationship to the ratio of neutral to acidic fractions in flaxseed gum obtained during this process. High separation temperatures increased the proportion of acidic fraction in flaxseed gum, thus, negatively affecting the viscosity, emulsification properties, and absorption capacity of the resultant mucilage.

Ding and Cui [104] discovered that dietary fibers make up 20% of the weight of flaxseed kernels, which are composed of both soluble and insoluble types. They separated five different kinds of soluble dietary fiber fractions on the basis of their solubility in various solvents in a sequential procedure and proposed that these soluble dietary fibers from flaxseed kernel can be utilized in different applications for attaining desirable viscosity without much thickening and added advantage of prebiotic and antioxidant activities. Fabre and Lacroux [105] tested three different physical methods for dietary fiber extraction from flaxseed, namely the microwave-assisted method, magnetic stirring, and ultrasound-assisted method. He found out that the ultrasound-assisted method with some magnetic stirring produced the best yields in a minimal time. The microwave method was not effective as it wasted a lot of energy. The ultrasound method decreased the viscosity of resultant mucilage to a small amount, which can be helpful for its further utilization.

Flaxseed mucilage and its novel forms, such as hydrogel and aerogel, can be used as a hydrocolloid in foods, cosmetics, pharmaceuticals, and biomaterials for obtaining various functional properties. FM is used in most applications for adding viscosity. At concentrations above 0.5%, FM exhibited shear thinning behavior where a decrease in viscosity was observed with the increasing amount of pressure, however at concentrations lower than 0.3%, FM exhibited non-Newtonian fluid behavior where its viscosity remained constant regardless of the amount of pressure applied [106]. These unique viscosity characteristics of FM are due to its two types of compositional fractions. The neutral fractions or arabinoxylans with large molecular weight polysaccharides gave shear thinning abilities to FM, whereas the acidic fractions with lower molecular weight sugars attributed non-Newtonian fluid behavior, which makes FM employable in a range of products [102].

The quality of flaxseed mucilage depends upon different factors during the extraction procedure; for example, high temperatures decrease the viscosity of FM by causing the breakdown of neutral polysaccharides into smaller acidic fractions along with protein denaturation [107]. Likewise, the pH conditions have a significant impact on the properties of FM. Under an acidic environment, the charges on building molecules of FM get suppressed, which decreases its viscosity. Whereas, under basic conditions, FM undergoes depolymerization, which also negatively affects its viscosity; therefore, FM with a sound structure and functional properties is obtained in a neutral pH zone (5–8 pH). Moreover, the presence of salt in a medium during the FM separation process decreased its viscosity by introducing repulsive forces and reducing the size of polysaccharide units [108]. FM has a greater solubility in water as compared to other plant-based gums. Flaxseed polysaccharides have comparable water binding and emulsification capacities with gum Arabica. The polar nature of sugars that compose FM is termed the reason for the wide range of its functional properties [16].

FM is increasingly being utilized as a steric stabilizer for salad dressings. The most stable emulsion of FM was obtained at a concentration of 0.75%, 2.5% salt, and pH = 4 [77]. Flaxseed mucilage is being successfully used as a meat binder due to its particular synergistic interactions with meat protein, thermal stability, desirable storage modulus, and gel stability, even under environments with greater salt percentages [109]. Flaxseed mucilage was found to significantly impact the quality of pork meat products by positively attributing to their moisture retention, yield, and texture [110]. Flaxseed gum, in combination with carrageenan and gellan gum, was utilized as a meat extender in meat sausages to replace starches and non-meat proteins. These improved the texture, color, stability, cohesiveness, water retention, and springiness of meat sausages and prevented fluid and fat losses during cooking to retain the quality of meat sausages [111]. FM has been utilized successfully as an additive in plant juices, dairy items, and flour products, where it contributed to the properties of thickness, emulsion stability, foam capacity, gelling ability, color, flavor, and nutrient retention [112,113].

Gel beads made from FM were employed for oil adsorption from the -water by Long, Zu [114], and it was revealed that their performance surpassed the abilities of activated carbon which is routinely being used for this purpose. FM also provides an additional advantage of being an environment-friendly, recyclable biomaterial than its other synthetic, non-degradable counterparts. FM is a good source of soluble dietary fiber, which has been found useful in the prevention of chronic ailments like diabetes, obesity, cardiovascular diseases, and even certain types of cancers like colon and rectal melanomas [115]. FM is increasingly being preferred by food manufacturers in their products over other food gums because it is required in much lesser quantity for obtaining the right viscosity and thus protects them from getting over-texturized [116].

### 3.5. Lignans

Lignans are macromolecules that exist in flaxseed, mainly in the form of SDG. The content of SDG in bread is influenced by various factors such as the amount of lignan or flaxseed meal added to the bread, the form of flaxseed used, and the type of bacterial or yeast cultures utilized in the leavening process. It was found that a significant quantity of SDG (73–75%), both in free and complex forms, was recovered from the samples, which indicated that SDG tolerated heat during the processes of milling, fermentation, and cooking [117]. In another study, Hyvärinen and Pihlava [46] observed the stability of SDG when added to both cold and hot dairy products such as milk, cheese, yogurt, and whey drinks. SDG withstood the temperature changes of fermentation, pasteurization, and refrigeration without much loss. Similarly, it was revealed that bread with added whole flaxseed and defatted flaxseed flour retained a high quantity of lignan content after processing steps of dough development, proofing, baking, and storage [118]. On the basis of evidence from these studies, it can be said that lignans undergo 10–25% recovery loss during cooking operations which can be further reduced by optimizing temperatures during heating treatments and studying the individual and combined impacts of all variables like flaxseed form, processing time, temperature, storage conditions, and microbial strains on the final SDG content of food products. Hyvärinen and Pihlava [46] reported that the SDG content of fermented foods like bread, cheese, and yogurt remained unaltered by starter bacterial strains like lactic acid bacteria even after long incubation times, which is a good sign that the desired nutritional benefits can be obtained from these fermented products. Microbial strains, which are routinely used in culturing of various food products, need to be characterized for their ability to retain or degrade lignan contents for maximizing functional advantage. Hall and Manthey [119] analyzed the thermal stability of flaxseed-fortified macaroni, which demonstrated that both the SDG and ALA content of the extruded product remained intact even after undergoing varying heat treatments during milling, drying, ultra-high temperatures, and shelf storage. The SDG depicted appreciable heat tolerability both in combined and separate forms. Shim and Olivia [120] produced gluten-free flour, dough, and bread with flaxseed meal and subjected them to storage study at various cold temperatures (4, −18, and −23 °C) and multiple sampling at 0, 1, 2, and 4 weeks intervals. The authors reported that the SDG content of all treatments remained constant throughout the entire study and unaffected by processing and storage temperatures.

## 4. Role of Flaxseed Compounds in the Maintenance of Gut Microbes and Human Health

**Figure 3:**
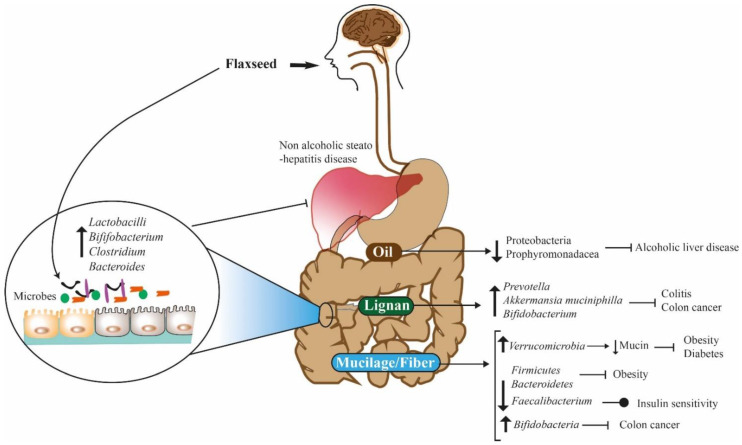
Effect of flaxseed components and gut health.

The gut biome is the microbial composition of human intestines, which plays a significant role in the maintenance of human health, immunity, cognitive functions, and the prognosis of different diseases. Flaxseed-derived compounds have been found to produce positive changes in the intestinal microbiota that help in the prevention and mitigation of various morbid conditions (Figure 3). The microbiome of a living being consists of trillions of microscopic organisms such as bacteria, viruses, yeasts, and protozoans [121]. *Bifidobacteria* is regarded as the most important genera that make up a large proportion of the intestinal flora of mammals. It is involved in the digestion and assimilation of the breakdown products of carbohydrate and lipid metabolism, which lead to beneficial changes in gut microbiota [122].

When flaxseed polysaccharides were subjected to in vitro fermentation, they generated a significant amount of xylose, arabinose, and rhamnose, which underwent further breakdowns to produce short-chain fatty acids like butyric or propionic acids [123]. These SCFAs are an important source of energy and anti-inflammatory action for various parts of the digestive tract. Propionate, butyrate, and acetate are produced inside the gut from the action of bacterial species like *Bifidobacteria longum* and *Eubacteria rectale* [124]. Flaxseed lignans are also transformed stepwise into their useful derivatives like secoisolariciresinol, enterodiol, and enterolactone by the action of various bacteria like *Bacillus*, *Clostridium*, *Klebsiella*, *Eubacterium*, *Peptostreptococccus*, *Ruminococcus*, *Nocardia*, and *Streptomyces* species, which then play an important role in human defense mechanisms against various diseases [125,126].

Beneficial changes in gut microbiota were linked with the consumption of polyunsaturated fatty acids in the human diet in many recent investigations [127]. It was found that the inclusion of flaxseed increased the percentage of *ω*-3 fatty acids, and prebiotic substances in the diet promoted an abundance of *Bifidobacteria* in the colon [128]. Different in vitro and in vivo models were employed by scientists to study the digestion of flaxseed components, which elucidated that the absorption process begins from the frontal portion of the small intestine [129,130]. Firstly, SCFAs are rapidly absorbed by intestinal epithelial cells into the blood, while medium and long-chain fatty acids are transported across them with the help of special proteins like fatty acid transport protein (FATP4), CD36, and fatty acid binding protein plasma membrane [131].

Fatty acids synthesized by gut bacteria are either utilized in the body for various processes or become part of triacylglycerol (TAG), the main energy storage molecules inside cell cytoplasm by the intricate enzymatic action of the endoplasmic reticulum. Some of the TAGs are later converted to chylomicrons by a multi-stage process inside the complex endoplasmic reticulum and Golgi apparatus, which then become part of systemic and lymphatic circulatory systems [132]. Gut bacteria also contribute to the metabolism of PUFA from ALA. The essential amino acid, alpha-linoleic acid (ALA), is situated at the *Sn*-2 position in TAG and cannot be hydrolyzed in living organisms, so it becomes the building block of other long-chain polyunsaturated fatty acids [133]. The absorption of ALA in the body by bacteria was greater in the emulsified form, which can contribute in a better manner towards metabolic processes [134].

Flaxseed substances have demonstrated their role in obesity prevention by making the desired changes in the gut microbiota of living beings. For example, there is a delicate balance of firmicutes and bacteriodes in our intestines; if the value of this ratio is high, it causes greater energy production from foods and increased storage of triglycerides in tissues [135]. Flaxseed mucilage reduces this F/B ratio by promoting the relative abundance of firmicutes along with the regulation of blood sugar levels, decreasing fat storage by suppressing the production of fasting-induced adipose factors, and contributing to satiety through its greater dietary fiber content, which altogether contributes to weight loss and obesity prevention [136,137]. The second mechanism through which flaxseed proteins were found to decrease weight among mice is by favoring the growth of certain bacterial species in cecal microbiota that have known roles in fat catabolism, such as *Bifidobacterium sp.* and *Akkermansia muciniphila* which are mucin degrading by nature, that prevent lipogenesis, fat deposition in liver and adipose tissue [137].

Another study has revealed the role of gut microbiota in bile acid metabolism, which can prevent fatty liver disease [138]. When mice were fed a flaxseed-enriched diet, it favored the growth of certain bacterial species, *Lactobacilli*, *Bifidobacterium*, *Clostridium*, and *Bacteroides*, which prevented the occurrence of non-alcoholic steatohepatitis disease by increasing the production of bile salt hydrolases and regulation of lipid metabolism via gut-axis pathway [139]. Similarly, various bacterial species from the genus Lactobacillus were analyzed for their effect on obesity prevention in multiple investigations, and it was discovered that *Lactobacillus gasseri* and *reuteri* inhibited free fatty acid absorption from intestines and lipid droplets formation in tissues that served as the reason for healthy weight loss [140,141,142].

Various aspects of the gut microbiome on the maintenance of human health have been reported, but the effect of flaxseed substances on their composition still needs to be discovered [5]. Flaxseed lignans are mainly metabolized by gut bacteria and transformed into mammalian lignans that perform a range of physiological functions inside the human body, out of which anti-cancer action is regarded as the most crucial one. Consumption of flaxseed brings a gradual shift in intestinal microbial composition by causing a greater prevalence of the bacteria that metabolize its polysaccharides, such as *Akkermansia*, *Bifidobacterium*, *Clostridium*, *Enterococcus*, *Lactobacillus*, *Megamonas*, *Phascolarctobacterium*, and *Prevotella species* [143]. These changes in gut bacteria have been found to play a role in decreasing inflammation and colitis, healing of gut lining, increasing insulin sensitivity, protecting against intestinal tumors, and slowing disease progression [144,145,146,147,148,149,150,151].

The gut microbiome has a huge influence on the mental well-being of a person as its bacterial residents are involved in the production of various substances, which are essential for nervous coordination, such as neurotransmitters, specific metabolites, brain-derived neurotrophic factors, short-chain fatty acids, tryptophan, GABA, etc. The dysregulation of those can lead to stress-related disorders, endocrine problems, and inflammatory diseases that occur when the homeostasis of these chemicals get disturbed from the negative changes in cecal bacterial composition [152,153,154,155,156]. The brain, nervous system, digestive system, and endocrine system are all interlinked through a system of sympathetic and para-sympathetic nerves, which constitute the brain–gut axis and have a direct impact on the normal physiological functioning of body organs and the immune system of mammals [153,157,158]. For example, flaxseed oil was found useful in the cure of polycystic ovarian syndrome among female rats due to the promotion of the growth of beneficial bacteria *Lactobacillus*, *Allobaculum*, *Desulfovibrio*, and *Bifidobacterium* and dis-favoring the growth of *Actinobacteria*, *Bacteroides,* Proteobacteria, and *Streptococcus*, which reduced the inflammation, disturbance of sex-steroid hormones, body weight, dyslipidemia, and insulin resistance through the gut–vaginal axis [159].

## 5. Conclusions and Future Perspective

Flaxseed is a nutrient and bioactive ingredient-rich plant crop. It possesses a high amount of fat, proteins, dietary fiber, lignans, vitamins, and minerals. The nutritional, functional, probiotic, and phytoactive properties of flaxseed are grabbing the attention of sensible consumers and food manufacturers alike. Flaxseed and its derived products can be utilized for a wide range of techno-functional purposes in the food industry, besides their newly discovered role in the regulation of intestinal microbiome and defense against various diseases. The consumption of flaxseed increases the probiotic bacteria in the gut and also produces metabolites that play crucial roles in lipid and glucose metabolism and immune and homeostasis pathways. The mechanisms by which flaxseed leads to positive changes in gut bacteria and improvement of various bodily functions need to be further investigated. The present investigation has also highlighted some areas that need to be worked upon to make the utilization of flaxseed bioactive compounds even better. (1) Flaxseed oil contains a large amount of polyunsaturated fatty acids, which are prone to rancidity during the later storage and processing stages. Thus it is necessary to find mechanisms for slowing down their oxidation and extending their shelf life. (2) Suitable emulsifiers need to be identified to improve the bioavailability of flaxseed components in microcapsules or emulsions forms. (3) Flaxseed oil is rich in linoleic and α-linoleic acids; it is important to trace the metabolic pathways which lead to the conversion of these fatty acids into essential fatty, eicosapentaenoic, and docosahexaenoic acids that are required for normal human body growth and functioning. (4) Mechanisms need to be researched by which flaxseed oil exerts various functional properties for improving the conversion rate to essential fatty acids and enhancing its gastrointestinal bioavailability. (5) Flaxseed lignans are converted into important mammalian lignans inside the human body by gut microbes that improve their absorption and bioavailability; it is necessary to find ways to further improve this conversion ratio, especially for lignan-fortified food products. (6) Flaxseed polysaccharides are a special research focus as they impart distinct functional properties to food when dissolved in solutions, such as the enhancement of viscosity, emulsification, gelation, and foaming properties, besides their known nutritional values. Methods need to be formulated for the production of FM biomaterials with consistent physicochemical and functional properties such as nano-gel, hydrogel, microgel, etc., and improve their overall digestion and absorption processes. (7) FM is a hydrocolloid; its properties and interaction with coexisting proteins need to be sorted out to make possible the delivery of bioactive compounds at the target sites. (8) Amino acid sequences of flaxseed proteins need to be determined, which can be helpful in generating peptides of desired nutritive purposes, biological activities, functional properties, and protease resistances that will make peptides maintain their integrity throughout the gastrointestinal tract. (9) Finally, more in vitro, in vivo, and nutritional interventional studies need to be conducted to assess the efficacy and efficiency of flaxseed products on the gut microbiome, human health, and protection against various diseases.

## Figures and Tables

**Table 2:** Food applications of flaxseed, its by-products, and bioactive compounds.

Flaxseed Form	Method of Processing/ Products Name	Dose of Flaxseed Supplementation	Mechanism	Reference
**Fortification of flaxseed in dairy products**				
Flaxseed lignan SDG	SDG stability in milk, yogurt, whey drinks, and cheese	1 g/10 L	↓SDG hydrolysis in cheese by the presence of lactic acid bacteria and enzymes. ↓SDG 25% due to the ↓pH of the whey drinks. ↑Temperature pasteurization of milk and whey. SDG was found stable.	[46]
HMPC and LMPI	Ice cream	0.5% and 1%	↑HMPC ↑Viscosity, LMPI did not affect the viscosity of ice cream; LMPI reduced sensory score more than gelatin. ↑Ice cream is overrun by ↑HMPC but ↓by higher LMPI. LMPI provides a better overrun than gelatin.	[47]
MEFOP	Fermentation or Indian yogurt	1–3%	↑The acidity of fortified yogurt samples may be due to lactose in the MFOP. ↑Gelling properties may be due to the capability of proteins to entrap without syneresis. ↑Peroxide value during storage due to FO, susceptible to oxidation.	[48]
FO	Ice cream	0–12%	↑Meltdown rate, ↓ice cream hardness linear to concentration.	[49]
Flaxseed as additive	Microstructure of flaxseed in butter	0.8–1.6%	Microstructure of flaxseed, globules, and cellular microstructure ↑butter structure, ↓degree of destruction.	[50]
**Fortification of flaxseed in baked products**				
Whole flaxseed flour	Bagels	30 g per bagel	Flax and grain ↑aroma and flavor, cinnamon raisin bagel ↓aroma and flavor, ↑sweet aroma and taste. Cinnamon raisin bagels had a higher acceptance rate of flavor compared to sunflower, sesame, and plain bagels.	[51]
Flaxseed meal and flour	Bagels and pretzel-type bakery products	5, 10, and 15%	The sample with 15% of flaxseed; ↓flavor 5% flaxseed ↓lightness or brightness values (*L*), whereas 10 and 15% flaxseed supplementation: ↓Fracture force, Formulation up to 10%; ↑crumb redness and darkness, has significant overall acceptability, nutritious and healthy substitute to consumers.	[52]
Flaxseed flour	Bread	15%	↓Loaf volume of bread, bright crust, and darker crumb	[53]
Flaxseed meal	Bread	15%	Flaxseed bread was evaluated during a period of 8 weeks in storage ↑crumb firmness, no significant differences in sensory attributes.	[54]
Raw and roasted ground flaxseed	Bread	10 g/100 g	Flaxseed enriched bread ↑water absorption, dough stickiness, and crumb softness. ↓Protein digestibility than the control.	[55]
Flaxseed hull extracts	Chinese steamed bread	1% (*w*/*w*)	↑Phytochemical content, ↑DPPH, ↑total phenolic content, ↑antioxidant activity.	[56]
Flaxseed flour	Bread	10, 15, 20, and 25%	Ground flaxseed 10%: ↑loaf volume, Dallman degree, nutritional content (linolenic acid and γ-tocopherol), and ↓staling brad. Flaxseed flour used with 15% ↑sensory acceptability.	[57]
Coated and uncoated ground flaxseed	Taftoon bread	5, 15, and 25%	↑Coated and uncoated ground flaxseed, ↓water absorption due to rich in oil can coat starch and gluten ↑stability, ↑dough development, and relaxation time. Ground flaxseed with Arabic gum ↑water absorption, ↑oxidative stability for 80 days at 25 °C.	[58]
Ground flaxseed	Yeast bread and muffins	15, 25, and 30%	Flaxseed improved the color of both bread and muffins due to the presence of leutin or zeaxanthin and the high protein content of flaxseed. 30% ground flaxseed appropriate formulations for bread.	[59]
Roasted flaxseed flour	Pan bread and pizza	10, 15, and 20%	Flaxseed with 15% ↑protein, ↑fat, ↑fiber, ↓carbohydrates, and ↓total serum cholesterol.	[60]
Full fat and partially defatted flaxseed flour	Unleavened flatbread	4, 8, 12, 16, and 20%	↑Acceptability of unleavened flatbreads with maximum flaxseed containing 12% full-fat flour and 16% partially defatted flour, ↑soluble and insoluble total dietary fiber, and ↑essential amino acids.	[61]
Flaxseed cake flour	Pita bread	5, 10, 15, and 20%	15 and 20% flaxseed: ↑Water absorption due to protein and mucilage, ↑mixing time (4.43 min) of dough, ↑extension (elasticity) of dough, ↑water holding capacity, ↑moisture content, ↑flaxseed cake flour, ↑alkaline water retention capacity.	[62]
Whole flaxseed flour	High Protein Cookies	12% (*w*/*w*)	↑Cookies hardness due to high protein, ↑darker and browner appearance, ↑sensory of 6 and 12% flaxseed, up to 12% flaxseed without negatively affecting the quality,	[63]
Golden flaxseed flour	Cereal bars	6, 12, and 18%	↑Nutritional qualities incorporated up to 12% without affecting their sensory and quality, ↑consumer acceptability, and no distinction between the control and 12% flaxseed cereal bars.	[64]
Roasted flaxseed, flour	Biscuits	10, 25, and 43%	10% flaxseed ↑quality (Moisture content, fortification, dark color, texture) and nutritional value without undesirable change.	[65]
Flaxseed flour	Biscuits	20, 30, and 40%	Flaxseed flour with 30% acceptable and 40% unacceptable, and product appearance was affected, i.e., the darker color and bitter taste were found by the panel.	[66]
Whole flaxseed flour	Muffin or snack bar	30 g per muffin or bar	↑Flax aroma, ↓sweetness, ↓vanilla aroma, ↑bitter taste, while no intensities on gingerbread raisin snack, ↑spice aroma, ↑nutritional value.	[67]
Flaxseed	Flaxseed boll	1 g per each boll	Flax balls under a cooking treatment balanced ω-3/ω-6 ratio, stable fatty acids profile, ↓CG, 16 days after anthesis bolls were more stable compared to 8 days after anthesis under heat treatment with good taste, texture, and aroma.	[68]
Defatted and non-defatted flaxseed flour	Wheat bread	10% NDF, 15% DF	DF and NDF fortified wheat flour: ↑protein, ↑fiber, ↑ash, ↓and carbohydrates, while 15% DF: ↓fat, ↑carbohydrates, ↑High-density lipoprotein-cholesterol, ↑triglycerides, ↓very low-density lipoprotein, ↓total cholesterol, ↓low-density lipoprotein.	[69]
Flaxseed flour	*ω*-3 rice paper	10% (*w*/*w*)	↑Antioxidant activity (231.7 mmol TE/g), ↑nutritional value.	[70]
Germinated and non-germinated flaxseed flour	Whole wheat bread	0, 5, 10, 15, and 20 % (*w*/*w*)	Germinated and non-germinated flaxseed: 10% acceptable for bread baking and sensory, ↑loaf volume, ↑and overall acceptability. Germinate flaxseed used in bread: ↑nutritional value, ↓decreased anti-nutrients ↑bioavailability, ↑nutritional absorption.	[71]
Whole flaxseed and crushed; FO and crushed flaxseed	Flaxseed bread roll and cinnamon roll	5% whole seed flour + 3% crushed seed, 13% FO + 1% crushed	In storage for 5 days at room temperature with no rancid odor detected, soft and remaining moisture content based on sensory panel and instrumental measurement.	[72]
Flaxseed flour	Corn snack	Up to 20%	↓Puffy extruded, probably due to protein and fat competition for water with starch	[73]
Ground flaxseed	Spaghetti	2.5–15%	↑Dough development time and strength, ↑dark color. Smaller flaxseed flour size, better food quality produced	[74]
Flaxseed flour	Muffins	2%, 5%	↑Flaxseed flour proportion, ↑viscosity, ↑Firmness, ↓elastic texture, ↑dark color with less redness, yellowness based on the Hunter scale, and no anti-staling effect.	[75]
Flaxseed flour	White bread	5–20%	Addition of flaxseed flour above 10%: ↑water absorption capacity, ↓dough stability and strength, ↑dough development time, ↑bread volume, ↓darker color of the crust, and ↑the value of crumb.	[76]
**Fortification of flaxseed in other products**				
FM extract	Salad dressing	0–1.5%	↑Protein content, ↑surface active properties, ↑emulsion stability at pH 6.0 and 2.0, ↑FM extract concentration in salad dressing, ↑viscosity, ↓and oil droplet size. Above mucilage concentration 0.45% (*w*/*w*), ↑stabilizing effect.	[77]
FP extract (high in protein and carbohydrate)	Potato dextrose agar	5%	Antifungal activity against (50%) *Penicillium* sp, *Fusarium gramineaum*, *Aspergillus flavus*, and 40% against *Pencillium chrysogenum* (under the conditions: 72 °C and 15 s), ↓acidic pH, ↑fungistatic activity.	[78]
Flaxseed extracts and meal	Pork meat	1.5% and 3%	↑Extended shelf life of meat, ↓oxidation of cholesterol and fatty acid, ↓peroxide value, and thiobarbituric acid-reactive substance.	[79]
Flaxseed cyclolinopeptides	Beef	100–200 µM	↓*Listeria monocytogene* activity during beef storage.	[80]
Flaxseed powder	Beef sausages	0,3, and 6%	↓Nitric content during storage, ↑linolenic acid, and no adverse effect on the sensory evaluation.	[81]
Flaxseed oil and extract	Liver pate	20% and 0.5–0.25%	↓Lipid oxidation, ↓monoenoic and saturated fatty acids, ↑polyene fatty acids, ↑phytosterols, and improve the oxidative stability of the product.	[82]

FO = flaxseed oil, FP = flaxseed protein, FM = flaxseed mucilage, SDG = secoisolariciresinol diglucoside, CG = gyanogenic glycoside, DF = defatted flaxseed, NDF = non-defatted, HMPC = high mucilage protein concentrate, LMPI = low mucilage protein isolate, MEFOP = microencapsulated FO powder.

## Data Availability

Not applicable.
